# Development of a Physiologically Based Pharmacokinetic (PBPK) Simulation Model for Nicotine

**DOI:** 10.1002/bdd.70018

**Published:** 2025-12-20

**Authors:** Brian Kim, Sung Hun Bae, Mohamed Bashar, Bofang Yi, Mohammad Asikur Rahman, Prince Awuah, Matthew Hartog, Rony Panarsky, Tao Zhang

**Affiliations:** ^1^ School of Pharmacy and Pharmaceutical Sciences Binghamton University State University of New York Binghamton New York USA; ^2^ Center for Tobacco Products, Division of Nonclinical Science Office of Science U.S. Food and Drug Administration Silver Spring Maryland USA

**Keywords:** GastroPlus, nicotine, pbpk simulation, pharmacokinetics

## Abstract

Tobacco is a major cause of chronic diseases such as lung cancer, cardiovascular disease, and chronic obstructive pulmonary disease worldwide. Nicotine, the primary psychoactive component in tobacco, is highly addictive and while not the primary driver of such tobacco‐related diseases, poses various health risks, particularly those affecting the cardiovascular and pulmonary systems. Although nicotine‐based therapies, such as nicotine replacement products, are widely utilized in smoking cessation efforts today, the impact of newer, tobacco delivery systems such as electronic nicotine delivery systems, or ENDS, remains uncertain and warrants continued evaluation. This study aims to develop and validate a physiologically based pharmacokinetic (PBPK) simulation model for nicotine using clinical pharmacokinetic data. The PBPK simulation model for nicotine was developed by incorporating drug‐specific and system‐specific parameters and by considering the systemic absorption, distribution, metabolism, and excretion of nicotine as well as its overall pharmacokinetic behavior on GastroPlus version 9.9. Validation of the developed PBPK model was performed by comparing predicted and observed plasma concentration–time profiles and pharmacokinetic parameters from clinical studies across multiple routes of administration including intravenous infusion, bolus, and pulmonary inhalation. The resulting model accurately captured plasma nicotine concentrations, with predicted pharmacokinetic parameters (*C*_max_, *T*_max_ and AUCs) falling within acceptable ranges of observed values and computational average fold error values. The current model provides a practical tool to translate systemic nicotine exposure across delivery systems, support dose optimization against predefined target exposure, and quantify safety margins, thereby informing safer product design and evidence‐based decisions in public‐health regulatory science.

## Graphical abstract

This study developed a region‐specific PBPK model for nicotine integrating various administration routes and lung compartments, validated using clinical PK data. This model enables an accurate prediction of nicotine kinetics and supports dose optimization for safer nicotine delivery systems.

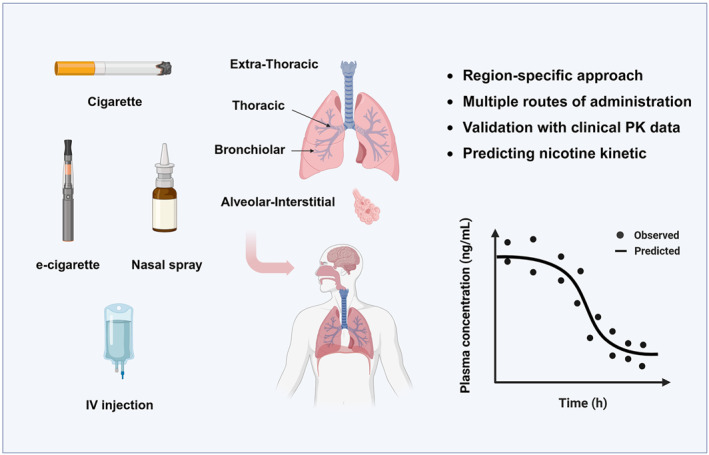

## 1 Introduction

Tobacco is the main cause of several chronic diseases including lung cancer, cardiovascular diseases, chronic obstructive pulmonary disease, and various other respiratory and vascular conditions. Although the smoking rate is declining in some regions due to public health campaigns for nonsmoking, tobacco remains a significant global health issue. It has been reported that tobacco is responsible for over 8 million deaths across the globe, with secondhand smoke exposure endangering the health of nonsmokers as well (He et al. 2022). Nicotine is an alkaloid compound in the tobacco plant, and it is the main psychoactive ingredient responsible for the addictive properties of tobacco (Sansone et al. 2023). Although electronic cigarettes, or e‐cigarettes, are often marketed as a safer alternative to traditional cigarettes, they continue to raise health concerns, and typically contain nicotine, contributing to their high potential for addiction (National Center for Chronic Disease Prevention and Health Promotion (US) Office on Smoking and Health 2016).

Nicotine is absorbed into the venous system of the lungs and is distributed to various organs and tissues including the liver, lungs, kidneys, and spleen (Benowitz et al. 2009). Once it reaches the brain, it binds to acetylcholine receptors, stimulating the release of neurotransmitters such as dopamine, leading to addiction (Jiloha 2010). Nicotine is primarily metabolized to cotinine in the liver via the CYP2A6 enzyme and is predominantly eliminated through the kidneys (Benowitz et al. 2009). The half‐life of nicotine is approximately 2 h in humans, while the half‐life of cotinine is substantially longer, at around 20 h, which is why it is often used as a biomarker for nicotine exposure (Benowitz et al. 2009).

Physiologically based pharmacokinetic (PBPK) simulation is a useful tool for predicting drug concentrations in both plasma and tissues (Miller et al. 2019). By integrating both drug‐specific properties with system‐dependent physiological and anatomical parameters, this modeling framework can account for interindividual variability across populations, including differences that arise from age, sex, ethnicity, and disease states. PBPK simulation modeling has seen rapid advancement in recent years and has played an increasingly important role in drug discovery and development across both academic and pharmaceutical sectors. Regulatory agencies such as the US Food and Drug Administration (FDA) and the European Medicines Agency (EMA) now recognize PBPK modeling as a standard tool to support regulatory decision‐making, the determination of doses, and the optimization of drug use in specific populations (Ladumor et al. 2019). Software platforms such as Simcyp (Certara, Sheffield, United Kingdom), GastroPlus (Simulation Plus Inc., Lancaster, CA, USA), PK‐Sim, and MOBI (Bayer Technology Services, Leverkusen, Germany) offer practical and validated examples of PBPK applications. Among these, GastroPlus has been widely used in pharmaceutical research, particularly in pharmacokinetics and pharmacodynamic (PK/PD) studies.

Recently, PBPK modeling has been widely used not only to support drug development but also to inform regulatory decision‐making, including dose optimization, assessment in special populations, and estimation of internal exposures. Despite this progress, there remains a lack of comprehensive and rigorously validated PBPK models specifically tailored for nicotine. This gap limits the ability to confidently apply PBPK modeling to regulatory evaluations and product development involving nicotine.

The objective of this study is to develop the PBPK model for nicotine that accurately simulates systemic and local tissue exposures across a range of exposure scenarios and delivery methods, with particular emphasis on respiratory tract deposition via the Pulmonary Compartmental Absorption & Transit (PCAT) model. The model we have built was constructed using physiological and compound specific parameters and validated using clinical pharmacokinetic data. By enabling quantitative estimates of nicotine distribution and clearance, this model serves as a robust platform for evaluating new nicotine formulations and conducting health risk assessments. Ultimately, it provides a scientifically grounded tool to support regulatory decisions, guide the development of new nicotine products, and help shape future research on nicotine exposure and safety.

## 2 Materials and Methods

### 2.1 Overview of PBPK Model Development for Nicotine

**FIGURE 1:**
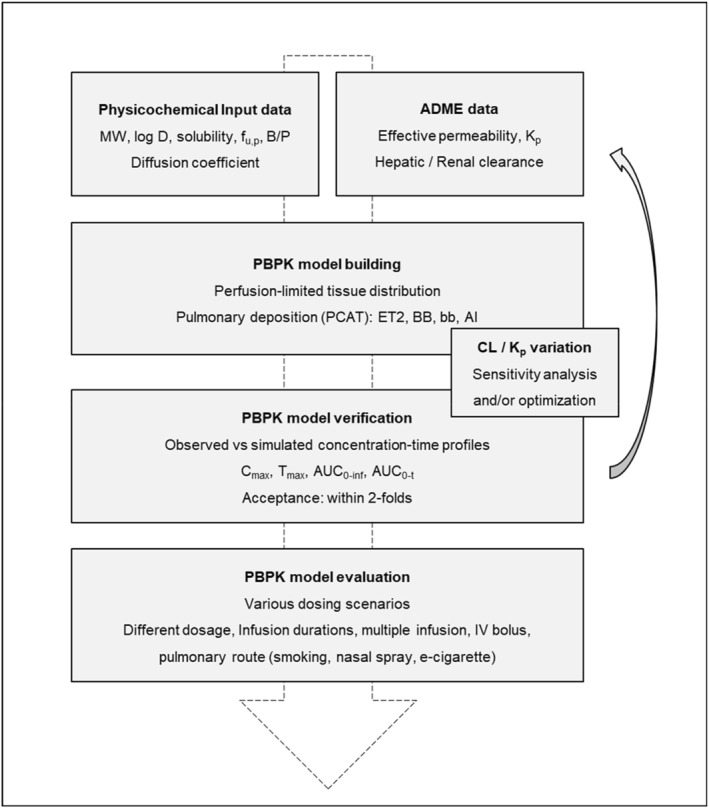
Schematic workflow for the development of nicotine physiologically based pharmacokinetic (PBPK) model.

The PBPK model for predicting nicotine plasma concentration was developed using GastroPlus version 9.9 (Simulation Plus Inc., Lancaster, CA, USA). The model was constructed based on a stepwise approach involving (1) collection of physicochemical and in vitro data, (2) PBPK model building and tissue distribution characterization, (3) parameter sensitivity analysis and optimization, (4) verification of model predictions using published clinical datasets, and (5) evaluation of model predictions for different administration conditions. A schematic representation of the model development process is shown in Figure 1.

### 2.2 Input Data and Parameterization

The PBPK model was constructed using input parameters derived from in vitro data, including the unbound fraction in plasma and in vitro intrinsic clearance. Additionally, ADMET Predictor version 11.0 (Simulation Plus Inc.) was used to estimate and optimize key physicochemical and absorption, distribution, metabolism, and excretion (ADME) properties such as the acid dissociation constant (pKa), pH‐dependent octanol‐water distribution coefficient (log D), permeability, solubility, and blood/plasma ratio, based on the molecular structure of nicotine. The parameters used for PBPK simulation are summarized in Table 1.

**TABLE 1:** Physicochemical properties and ADME of nicotine for PBPK simulation.

Parameter	Input value	Reference/comment
Physicochemical properties		
Molecular weight (g/mol)	162.24	ADMET
Log D	1.1	ADMET
Solubility (mg/mL)	93.3 (at pH 7.4)	Drug bank
Human *f*_u,p_ (%)	95.0	Benowitz et al. (2009)
Human B/P ratio	1.2	ADMET
Diffusion coefficient (cm^2^/s × 10^5^)	0.0846	Asgharian et al. (2018)
Drug particle density (g/mL)	1.2	Default
Absorption		
Effective permeability (cm/s × 10^4^)	4.28	ADMET
Distribution		
*K*_*p*_	Table S3	Optimized
Elimination		
Module 1 (CL‐based model)		
CL (L/h) in liver	40	Optimized
CL (L/h) in kidney	10	Optimized
Module 2 (CYP2A6 enzyme kinetic)		
*V*_max_ (nmol/min/pmol CYP)	0.011	Yamazaki et al. (1999)
*K*_*m*_ (μmol/L)	11.0	
VISEF	0.36	

Abbreviations: B/P ratio, the blood‐to‐plasma partition *ratio*; CL: the clearance; *f*_u,p_, the unbound fraction of drugs in plasma; *K*_*m*_, the concentration at which half of *V*_max_ for the metabolism of nicotine; *K*_*p*_, the tissue to plasma partition coefficients; LogD, the distribution of a compound between the aqueous and lipid phases; *V*_max_, the maximum velocity of reaction; VISEF, Intersystem Extrapolation Factor.

### 2.3 Stepwise Model Construction

#### 2.3.1 Model Development

The model was built using physicochemical and clearance data from in vitro and in silico sources. Demographic characteristics such as age, weight, and body mass index (BMI) range for each simulation were obtained from the corresponding clinical trials (Supporting Information S1: Table S1). The simulated population as configured on GastroPlus represented healthy American individuals, with a 50% male‐to‐female ratio, and without incorporation of additional physiological conditions such as renal impairment, cirrhosis, steatosis, nor pregnancy. In cases where specific population parameters were not available from the literature, default values provided by GastroPlus were used. The absorption of nicotine was developed based on the parameters generated from ADMET Predictor (Simulations Plus Inc.). Tissue distribution was developed using a perfusion‐limited approach, reflecting nicotine's high permeability. Metabolism and excretion were modeled to occur primarily through the liver and kidneys, respectively. For elimination, the organ clearances and tissue to plasma partition coefficients (*K*_*p*_) were adjusted within physiologically reasonable ranges to achieve agreement between observed and simulated systemic exposure (Supporting Information S1: Table S2). The elimination was built with two approaches. The first method, hepatic clearance (CL_H_) and renal clearance (CL_R_) clearances were applied. In the second method, hepatic clearance was estimated using CYP2A6 enzyme kinetic parameters which were scaled to whole‐liver metabolism using an intersystem extrapolation factor (VISEF) adjusted empirically to improve the simulation fit. The hepatic metabolism of nicotine was assumed to be exclusively mediated by CYP2A6, whereas the CL_R_ accounted for approximately 20% of the total systemic clearance. Accordingly, the CL_H_ was set to 80% of the total clearance (CL), and CL_R_ was calculated inversely based on this assumption. The simulation of nicotine for inhalation administration was conducted with detailed local deposition of drug absorption in various regions of the lung combined with systemic drug distribution and elimination using the nasal‐pulmonary mechanistic model in GastroPlus as described below. Both approaches were evaluated by comparing model performance with observed pharmacokinetic data.

#### 2.3.2 Pulmonary Absorption Modeling

To quantify the deposition of nicotine in the respiratory tract following traditional smoking, nasal spray, and e‐cigarette administration, the dosimetry model that partitions the lung into specific anatomical regions was applied. This Pulmonary Compartmental Absorption and Transit (PCAT) model consists of the nasal region (for nasal spray), and compartmentalization of the respiratory tract into the extra‐thoracic (ET2), thoracic (BB), bronchiolar (bb), and alveolar‐interstitial (AI) regions. The percentage depositions of inhaled nicotine along these regions of the respiratory tract were allocated based on previously published studies which describe the regional distribution of inhaled nicotine as follows: approximately 20% in the buccal cavity (BC), 25% in the upper respiratory tract (URT), 50% in the lower respiratory tract (LRT), and 5% exhaled (Rostami et al. 2022). The ET2 region is mainly comprised of the posterior nasal passages, larynx, pharynx, and mouth; the BB region by the trachea and bronchi; the bb region up to the terminal bronchioles; and the AI region by the respiratory bronchioles, alveolar duct, and alveoli (Belete and Msganaw Shiferaw 2023). Using this anatomical delineation of where each compartment of the PCAT model starts and ends, the aforementioned percentage deposition of nicotine from Rostami et al. and related studies were allocated accordingly. The specific values entered into GastroPlus based on this framework are presented in Supporting Information S1: Table S3.

#### 2.3.3 Sensitivity Analysis

Sensitivity analysis was performed for CL_H_, plasma unbound fraction (*f*_u,p_), and hematocrit to identify parameters with the greatest influence on model outputs. Based on the sensitivity analysis, the parameters related to distribution and elimination (*K*_*p*_, liver and kidney clearance) were subsequently optimized to improve the predictive performance of the model. This adjustment allowed the simulated concentration–time profiles to more closely match the observed data and improved the overall reliability of the model.

#### 2.3.4 PBPK Model Verification

The accuracy of our PBPK model was validated by comparing simulated plasma concentration–time profiles and pharmacokinetic parameters against clinical data reported in literature. These parameters, obtained either directly from literature or calculated using the PKPlus module embedded in GastroPlus, included area under the curve (AUC), maximum plasma concentration (*C*_max_), and time to reach *C*_max_ (*T*_max_). Observed concentration–time profiles were digitized using WebPlotDigitizer version 4.7 (https://automeris.io/v4/) with the corresponding time points serving as a reference for evaluating the goodness of fit between the extrapolated observed values and our model's simulated outputs. Model predictions were considered acceptable when simulated parameters were within a two‐fold range of observed values, denoted by the shaded region between dashed lines.

#### 2.3.5 PK Model Evaluation for Various Administration Conditions

Predicted plasma concentration–time profiles and PK parameters were compared against multiple clinical studies and across several different routes of administration, infusion times, and dose levels to evaluate our model's predictive performance. This includes intravenous (IV) bolus, smoking, nasal spray, and e‐cigarettes. Studies were selected based on data quality, availability of complete concentration–time profiles, and relevance to commonly used nicotine delivery systems. The model was deemed satisfactory when simulated *C*_max_, *T*_max_, and AUC values were within a two‐fold difference of observed data, as recommended by current PBPK verification practices.

For each of the aforementioned routes, simulations were customized to reflect route‐specific input parameters such as bioavailability, absorption rate, and deposition site. For example, inhalation routes such as smoking and e‐cigarette use incorporated estimates of lung deposition and absorption kinetics, whereas intravenous administration assumed 100% bioavailability with immediate systemic entry. Dose levels, infusion durations, and puffing topography were aligned with reported study protocols to ensure an accurate representation of practical scenarios. In the case of e‐cigarettes, simulations were conducted using an e‐liquid concentration of 18 mg/mL, corresponding to an estimated 0.14 mg of nicotine delivered per puff. A standard usage pattern of 10 puffs per session, spaced at 30‐s intervals, was modeled, with two total sessions occurring one hour apart (Rostami et al. 2022). Because of the short time intervals and exposure duration, 100% of the inhaled nicotine dose was assumed to be absorbed, without accounting for exhalation losses. Based on this assumption, the absorbed dose per puff was 0.14 mg, resulting in a total nicotine intake of 1.4 mg per session (0.14 mg/puff ☓ 10 puffs). Similar modeling inputs were used for traditional smoking scenarios, where puff count, interval, and puff volume were defined based on study‐specific puffing regimens.

### 2.4 Statistical Analysis

All simulated PK parameters (*C*_max_, *T*_max_, AUC_0‐inf,_ and AUC_0‐t_) were presented as geometric means (maximum ∼ minimum). The calculation of the *R* ratio, comparing observed and simulated pharmacokinetic parameters, was performed by the following equation.

$$R\;\text{ratio}=\frac{\text{Observed}\;\text{PK}\;\text{parameter}}{\text{Predicted}\;\text{PK}\;\text{parameter}}$$

The *R* ratio between 0.5 and 2.0 was considered acceptable (Zamir et al. 2023). In addition, the average fold error (AFE), root mean squared error (RMSE), and mean absolute error (MAE) for the pharmacokinetic (PK) parameters (*C*_max_, *T*_max_, AUC_0‐inf_ and AUC_0‐t_) were calculated to improve the model assessment. The equations are shown below.

$$\text{Fold}\;\text{error}=\frac{Predicted\;parameter\;value}{Observed\;parameter\;value}$$

$$\text{AFE}=10^{\frac{\sum \log\left(fold\;error\right)}{N}}$$

$$\text{RMSE}=\sqrt{\frac{1}{n}*\sum {\left(simulated_{i}-observed_{i}\right)}^{2}}$$

$$\text{MAE}=\frac{1}{n}*\sum \left|simulated_{i}-observed_{i}\right|$$

## 3 Results

### 3.1 Establishment of the Nicotine PBPK Model After Intravenous Infusion

**FIGURE 2:**
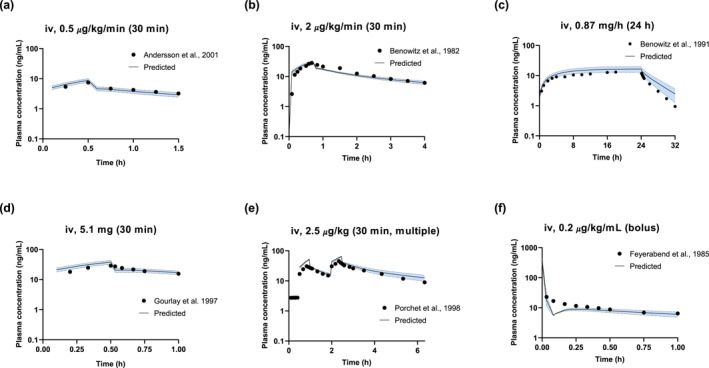
Plasma concentration–time profiles after IV administration of nicotine. Figures (a–e) present data on IV infusion and figure (f) shows data related to IV bolus. The observed data were shown by circle symbol, while the predicted data were represented by a solid line. The shadow region represented predicted standard deviation. IV, intravenous.

The simulation results after intravenous administration of nicotine were compared with the pharmacokinetic study reported (Figure 2). The parameters used for PBPK simulation are summarized in Table 1. The total clearance of nicotine used in the model was close to literature‐reported values (Olsson Gisleskog et al. 2021), as were our optimized values for *K*_*p*_ and volume of distribution at steady state (V_ss_). Since the liver was responsible for metabolizing approximately 80% of nicotine clearance according to previous research findings (Scharf et al. 2022), in the model the total body clearance was divided into two portions, with 80% attributed to the liver and 20% attributed to the kidney. The values for *K*_*p*_ for different organs and tissues are outlined in Supporting Information S1: Table S2, showing distribution to be the highest in the lung, liver, kidney, heart, and spleen, and low in adipose tissue. Such findings are consistent with human autopsy samples from smokers as reported by Hukkanen et al. (2005). Similarly, the V_ss_ was 2.70 L/kg, which fits within the literature reported range of 1.8–3.3 L/kg (Olsson Gisleskog et al. 2021). From these values, the terminal half‐life of nicotine predicted by our model was 2.85 h, which is similar with the reported t_1/2_ of 2–3 h in the human plasma (Benowitz et al. 1991).

The observed and predicted mean plasma areas under the curve extrapolated to infinity (AUC_inf_) were 13.2 and 13.7 ng·h/mL, respectively. The observed and predicted mean plasma areas under the curve from time zero to time t (AUC_0‐t_) and maximum plasma concentration (*C*_max_) for nicotine were 6.76 and 7.07 ng·h/mL, and 7.46 and 8.80 ng/mL, respectively. This gives us a fold‐error of 1.04 for AUC_inf_, 1.05 for AUC_0‐t_, and 1.18 for *C*_max_. The resulting plasma concentration profiles were plotted alongside observed values for visual comparison and showed strong agreement.

**FIGURE 3:**
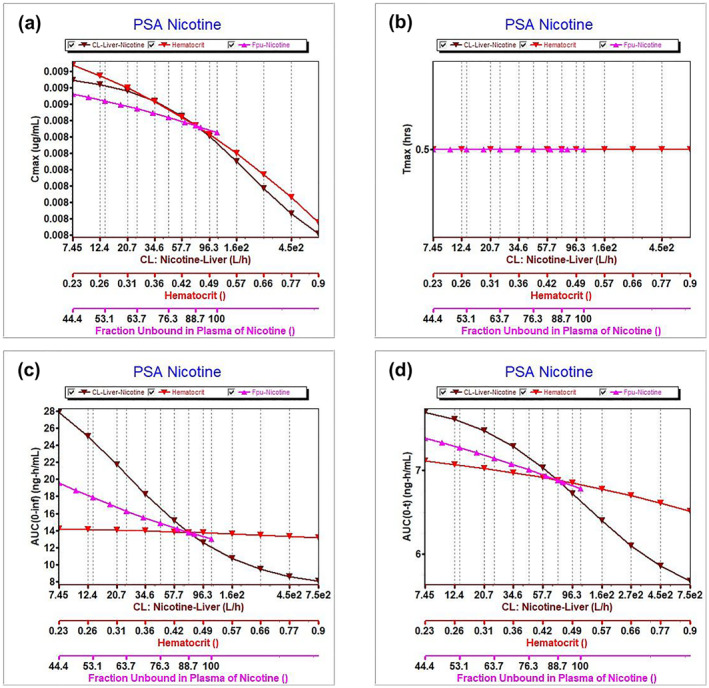
Parameter sensitivity analysis for CL in liver, hematocrit, and fraction unbound in plasma of nicotine on (a) *C*_max_ (b) *T*_max_ (c) AUC_0‐inf_ (d) AUC_0‐t_ for nicotine. *C*_max_, the maximum plasma concentration; *T*_max_, the time to reach the maximum plasma concentration; AUC_0‐inf_, the mean plasma areas under the curve extrapolated to infinity; AUC_0‐t_, the observed and predicted mean plasma areas under the curve from time zero to time t; CL, clearance.

Sensitivity analysis was performed to hepatic clearance, plasma unbound fraction, and hematocrit (Figure 3). This is because nicotine is mainly metabolized by liver, with renal excretion contributing less than 20% of total clearance (Scharf et al. 2022). Moreover, *K*_*p*_ values mainly affect tissue distribution rather than systemic exposure (*C*_max_ and AUCs), and simultaneous variation with *K*_*p*_ may complicate model convergence. Therefore, the sensitivity analysis was focused on physiologically relevant factors for systemic pharmacokinetics.

We further developed a model by incorporating CYP2A6 enzyme kinetic parameters to mechanistically represent hepatic metabolism. The model showed pharmacokinetic profiles comparable to the current model and observed data (Supporting Information S1: Figure S1 and Table S4). Predicted *C*_max_, *T*_max,_ and AUCs values were consistent with those of the current model. Additionally, the CYP2A6 enzyme kinetic model reproduced the plasma concentration–time profiles and exposure parameters of nicotine within experimental variability, suggesting that enzyme kinetic incorporation did not alter model performance but also could provide a mechanistic representation of hepatic metabolism.

### 3.2 Nicotine PBPK Model Development After Intravenous and Inhalation Dosing Routes

The validated model, developed and verified through intravenous infusion study, was subsequently applied to evaluate and predict the nicotine pharmacokinetics for various doses and routes of administration. Figure 2 shows the observed and simulated plasma concentration–time profiles of nicotine following intravenous infusion and bolus administration. The peak concentration of nicotine should be reached at the time when infusion stopped (Urso et al. 2002); however, there was a mismatch of the observed PK profile with the clinical protocol. Therefore, the infusion time was adjusted based on the observed results during the PBPK simulation development if it was not clearly indicated on the graph.

**FIGURE 4:**
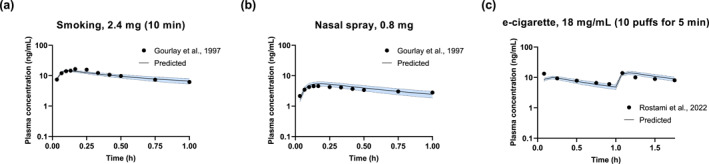
Plasma concentration–time profiles after the pulmonary route of nicotine administration. (a) smoking; (b) nasal spray; (c) e‐cigarettes. The observed data were shown by a circle symbol, whereas the predicted data were represented by a solid line. The shadow region represented predicted standard deviation. e‐cigarettes, Electronic cigarettes.

**FIGURE 5:**
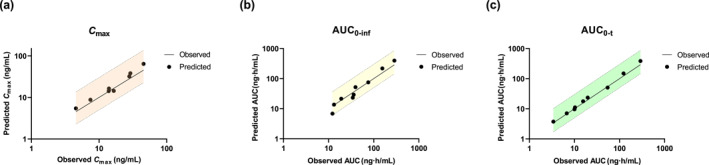
Comparison of observed and predicted pharmacokinetic parameters (*C*_max_ (a), AUC_0‐inf_ (b) and AUC_0‐t_ (c)) for nicotine. The closed circles are the predicted values and black dashed lines represent 2‐fold deviation. The result of *C*_max_ after IV Bolus administration is not shown in the graph. *C*_max_, the maximum plasma concentration; AUC_0‐inf_, the mean plasma areas under the curve extrapolated to infinity; AUC_0‐t_, the observed and predicted mean plasma areas under the curve from time zero to time t.

The comparison of the model‐predicted main pharmacokinetic parameters (*C*_max_, *T*_max_, AUC_inf,_ and AUC_0‐t_) with the observed data and the calculated fold errors for these parameters are summarized in Tables 2 and 3. For the inhalation route of nicotine administration, the model was enhanced to include pulmonary absorption dynamics and deposition patterns within the respiratory tract. For the e‐cigarettes model, simulation with an e‐liquid concentration of 18 mg/mL predicted the nicotine delivery of 0.14 mg per puff, resulting in a total intake of 1.4 mg per session (10 puffs). Assuming complete absorption without exhalation loss, this pattern was comparable to traditional smoking scenarios modeled using study‐specific puffing parameters. It has been reported that an *R* ratio for PK parameters within a 2‐fold difference between the observed and simulated data is acceptable from the industry's perspective (Lee et al. 2019). Hence, our predicted results were deemed reasonable by comparison to clinical trial data according to this standard. As can be seen in Figures 2 and 4, the predicted plasma concentration–time profiles of nicotine were in close agreement with the clinically observed profiles from their respective studies. In addition, all the fold errors for parameters, *C*_max_, *T*_max_, AUC_inf,_ and AUC_0‐t_, were between 0.5 and 2.0 of the observed data, with 100% of AUCs and *C*_max_ *R* ratios falling within the 1.5‐fold difference (Figure 5). This indicates a high degree of model accuracy and supports the robustness of the model's predictive performance across key pharmacokinetic endpoints.

**TABLE 2:** The arithmetic mean (min ∼ max) of observed and predicted pharmacokinetic parameters for nicotine after intravenous administration.

Dose	Routes/Time	Parameters	Observed	Predicted	*R* Ratio	Reference
0.5 *μ*g/kg/min	Infusion 30 min	*C*_max_ (ng/mL)	7.46	8.80 (7.59–11.0)	0.848	Andersson and Arner (2001)
		*T*_max_ (h)	0.500	0.500	1.00	
		AUC_0‐inf_ (ng·h/mL)	13.2	13.7 (10.3–21.4)	0.967	
		AUC_0‐t_ (ng·h/mL)	6.76	7.07 (5.91–8.49)	0.957	
2 *μ*g/kg/min	Infusion 30 min	*C*_max_ (ng/mL)	28.0	32.1 (29.2–39.0)	0.873	Benowitz et al. (1982)
		*T*_max_ (h)	0.667	0.700	0.953	
		AUC_0‐inf_ (ng·h/mL)	75.7	75.4 (61.8–91.0)	1.00	
		AUC_0‐t_ (ng·h/mL)	54.4	50.6 (46.5–56.7)	1.08	
0.87 mg/hr	Infusion 24 h	*C*_max_ (ng/mL)	14.0	16.4 (11.0–22.8)	0.854	Benowitz et al. (1991)
		*T*_max_ (h)	17.3	24.0	0.721	
		AUC_0‐inf_ (ng·h/mL)	287	398 (264–551)	0.721	
		AUC_0‐t_ (ng·h/mL)	291	385 (260–529)	0.756	
5.1 mg	Infusion 30 min	*C*_max_ (ng/mL)	29.0	38.1 (30.3–51.2)	0.761	Gourlay and Benowitz (1997)
		*T*_max_ (h)	0.500	0.500	1.00	
		AUC_0‐inf_ (ng·h/mL)	39.5	52.1 (39.2–66.2)	0.758	
		AUC_0‐t_ (ng·h/mL)	19.7	23.5 (19.2–31.5)	0.838	
2.5 *μ*g/kg	Multiple infusion 30 min	*C*_max_ (ng/mL)	45.4	64.2 (58.8–66.6)	0.708	Porchet et al. (1988)
		*T*_max_ (h)	2.33	2.45	0.951	
		AUC_0‐inf_ (ng·h/mL)	155	218 (174–269)	0.710	
		AUC_0‐t_ (ng·h/mL)	123	149 (132–167)	0.827	
0.2 *μ*g/kg/mL	Bolus	AUC_0‐inf_ (ng·h/mL)	34.4	23.3 (17.5–30.7)	1.48	Feyerabend et al. (1985)
		AUC_0‐t_ (ng·h/mL)	10.3	11.1 (10.2–13.1)	0.928	

Abbreviations: AUC_0‐t_, the observed and predicted mean plasma areas under the curve from time zero to time t; AUC_0‐inf_, the mean plasma areas under the curve extrapolated to infinity; *C*_max_, the maximum plasma concentration; *T*_max_, the time to reach the maximum plasma concentration.

**TABLE 3:** The arithmetic mean (min ∼ max) of observed and predicted pharmacokinetic parameters for nicotine after the pulmonary route administration.

Dose	Routes/Time	Parameters	Observed	Predicted	R Ratio	Reference
2.4 mg	Smoking 10 min	*C*_max_ (ng/mL)	16.5	14.4 (13.2–15.8)	1.14	Gourlay and Benowitz (1997)
		*T*_max_ (h)	0.167	0.111 (0.100–0.133)	1.50	
		AUC_0‐inf_ (ng·h/mL)	19.1	21.4 (14.5–27.8)	0.894	
		AUC_0‐t_ (ng·h/mL)	10.0	9.64 (8.15–11.3)	1.04	
0.8 mg	Nasal spray	*C*_max_ (ng/mL)	4.55	5.45 (4.45–6.60)	0.835	Gourlay and Benowitz (1997)
		*T*_max_ (h)	0.167	0.184 (0.167–0.200)	0.910	
		AUC_0‐inf_ (ng·h/mL)	12.2	6.85 (5.09–9.80)	1.78	
		AUC_0‐t_ (ng·h/mL)	3.41	3.74 (2.88–4.90)	0.911	
18 mg/mL	E‐cigarette 10 puffs with a 30 s interval	*C*_max_ (ng/mL)	13.9	14.2 (11.4–18.5)	0.976	Gourlay and Benowitz (1997)
		*T*_max_ (h)	1.08	1.17	0.926	
		AUC_0‐inf_ (ng·h/mL)	35.9	29.3 (20.8–37.8)	1.22	
		AUC_0‐t_ (ng·h/mL)	15.6	17.7 (13.5–23.5)	0.883	

Abbreviations: AUC_inf_, the mean plasma areas under the curve extrapolated to infinity; AUC_0‐t_, the observed and predicted mean plasma areas under the curve from time zero to time t; *C*_max_, the maximum plasma concentration; *T*_max_, the time to reach the maximum plasma concentration.

Additionally, the predictive accuracy of the developed PBPK model for nicotine was quantitatively assessed using the average fold error (AFE), root mean squared error (RMSE), and mean absolute error (MAE) calculated from observed and simulated PK parameters (Tables 4 and 5). For all intravenous infusion and bolus administration scenarios, the AFE values for *C*_max_, *T*_max_, AUC_0‐t,_ and AUC_0‐inf_ were close to unity (1.00–1.13). The RMSE and MAE values were also within acceptable ranges, supporting the reliability of the model's predictive performance across various dosing regimens. Similarly, good predictive performance was observed for pulmonary routes, including smoking, nasal spray, and e‐cigarette. The AFE values for these conditions ranged from 1.00 to 1.03, whereas RMSE and MAE values remained low in all PK parameters, indicating minimal deviation between observed and simulated nicotine concentrations. These results suggest that the PBPK model was adequately established with the systemic pharmacokinetic profiles of nicotine following both intravenous and pulmonary administrations. Overall, the AFE values were close to 1.00 and the small RMSE and MAE values in the results show that the developed PBPK model is more reliable and could accurately describe nicotine exposure following different types of administration.

**TABLE 4:** The computational average fold error values for PK parameters for nicotine after intravenous administration.

Dose	Routes/Time	Parameters	AFE	RMSE	MAE
0.5 *μ*g/kg/min	Infusion 30 min	*C*_max_ (ng/mL)	1.01	1.16	1.03
		*T*_max_ (h)	1.00	0.00	0.00
		AUC_0‐inf_ (ng·h/mL)	1.02	2.93	2.04
		AUC_0‐t_ (ng·h/mL)	1.01	0.918	0.838
2 *μ*g/kg/min	Infusion 30 min	*C*_max_ (ng/mL)	1.00	2.72	2.56
		*T*_max_ (h)	1.00	0.00	0.00
		AUC_0‐inf_ (ng·h/mL)	1.01	9.55	7.48
		AUC_0‐t_ (ng·h/mL)	1.00	2.25	1.73
0.87 mg/hr	Infusion 24 h	*C*_max_ (ng/mL)	1.13	3.47	2.99
		*T*_max_ (h)	1.00	0.00	0.00
		AUC_0‐inf_ (ng·h/mL)	1.13	85.3	73.4
		AUC_0‐t_ (ng·h/mL)	1.13	79.0	68.4
5.1 mg	Infusion 30 min	*C*_max_ (ng/mL)	1.01	5.50	4.28
		*T*_max_ (h)	1.00	0.00	0.00
		AUC_0‐inf_ (ng·h/mL)	1.01	6.88	5.21
		AUC_0‐t_ (ng·h/mL)	1.01	3.31	2.57
2.5 *μ*g/kg	Multiple infusion 30 min	*C*_max_ (ng/mL)	1.00	2.66	2.34
		*T*_max_ (h)	1.00	0.00	0.00
		AUC_0‐inf_ (ng·h/mL)	1.01	36.7	34.1
		AUC_0‐t_ (ng·h/mL)	1.01	13.8	13.0
0.2 *μ*g/kg/mL	Bolus	AUC_0‐inf_ (ng·h/mL)	1.02	4.97	4.30
		AUC_0‐t_ (ng·h/mL)	1.01	1.10	0.946

Abbreviations: AFE, average fold error; AUC_0‐inf_, the mean plasma areas under the curve extrapolated to infinity; AUC_0‐t_, the observed and predicted mean plasma areas under the curve from time zero to time t; *C*_max_, the maximum plasma concentration; MAE, mean absolute error; RMSE, root mean squared error; *T*_max_, the time to reach the maximum plasma concentration.

**TABLE 5:** The computational average fold error values for PK parameters for nicotine after the pulmonary route administration.

Dose	Routes/Time	Parameters	AFE	RMSE	MAE
2.4 mg	Smoking 10 min	*C*_max_ (ng/mL)	1.00	0.788	0.583
		*T*_max_ (h)	1.01	0.0156	0.0147
		AUC_0‐inf_ (ng·h/mL)	1.03	5.22	4.88
		AUC_0‐t_ (ng·h/mL)	1.01	1.04	0.890
0.8 mg	Nasal spray	*C*_max_ (ng/mL)	1.01	0.817	0.762
		*T*_max_ (h)	1.01	0.0165	0.0165
		AUC_0‐inf_ (ng·h/mL)	1.02	1.53	1.21
		AUC_0‐t_ (ng·h/mL)	1.01	0.656	0.546
18 mg/mL	E‐cigarette 10 puffs with a 30‐s interval	*C*_max_ (ng/mL)	1.00	1.69	1.31
		*T*_max_ (h)	1.00	0.00	0.00
		AUC_0‐inf_ (ng·h/mL)	1.01	4.47	3.56
		AUC_0‐t_ (ng·h/mL)	1.01	2.43	1.91

Abbreviations: AFE, average fold error; AUC_0‐inf_, the mean plasma areas under the curve extrapolated to infinity; AUC_0‐t_, the observed and predicted mean plasma areas under the curve from time zero to time t; *C*_max_, the maximum plasma concentration; MAE, mean absolute error; RMSE, root mean squared error; *T*_max_, the time to reach the maximum plasma concentration.

## 4 Discussion

Nicotine, widely known for its addictive properties, is the main psychoactive component in tobacco products, influencing the central nervous system and increasing tobacco dependence (Sansone et al. 2023). Recently, nicotine replacement therapies have been used to aid smoking cessation by alleviating withdrawal symptoms, but nicotine exposure is still associated with cardiovascular effects and has been implicated in cancer biology, though a direct causal link to cancer progression remains unproven (Benowitz et al. 2009; Benowitz and Burbank 2016; Kim et al. 2023; Mishra et al. 2015). Effective management of nicotine is therefore crucial due to its addictive nature and potential interactions with other medications, particularly in patients undergoing multiple drug therapies.

The main purpose of this work is to develop the PBPK model of nicotine using GastroPlus, which can be used to estimate systemic nicotine exposure and its effects on organs of the respiratory tract. One distinguishing feature of our PBPK model is the use of the nasal‐pulmonary module embedded in GastroPlus, which enables deposition tracking across anatomically distinct regions: extra‐thoracic, bronchiolar, thoracic, and alveolar‐interstitial. Unlike earlier studies that used broader classifications such as the buccal cavity, the upper respiratory tract, and the lower respiratory tract, this region‐specific approach improves the physiological accuracy of pulmonary absorption modeling and reflects a more mechanistic understanding of site‐specific deposition. The resulting PBPK model provides a more anatomically granular and comprehensive method of describing nicotine's systemic distribution and pulmonary absorption following intravenous and inhalation administrations. Such PBPK simulations could support the development of innovative nicotine delivery systems and contribute to understanding the broader health implications of nicotine.

It has also been noted that increased tissue retention and plasma protein binding driven by physiological changes could lead to differential consumption of combustible cigarettes (Kolli 2023). Deviations in our PBPK model, as shown in Figures 2 and 4, may be the result of differences in physiology from choosing a healthy population when configuring our model on GastroPlus. This excludes the possibility of physiological changes due to conditions like cirrhosis, renal impairment, and pregnancy, which may exist in the true population. The predicted *C*_max_ was a little higher than observed data after intravenous infusion administration (Figure 2). Despite these differences, however, the reliability of our model in predicting the plasma concentration of nicotine is still deemed reasonable when quantifying the differences in PK parameters through the calculation of associated *R* ratios (Lee et al. 2019). Additional simulations using the CYP2A6 enzyme kinetic model were performed to mechanistically represent the hepatic metabolism of nicotine. Incorporating enzyme kinetic parameters derived from recombinant CYP2A6 allowed the assessment of whether in vitro kinetic information could reproduce the in vivo pharmacokinetics observed in humans. The model generated PK parameters that were comparable to those of the current clearance‐based model and consistent with the observed plasma concentration–time data (Supporting Information S1: Figure S1 and Table S4). The enzyme kinetic approach provides a flexible and mechanistic framework that could be extended to evaluate interindividual variability or genetic polymorphisms in CYP2A6 activity, which are known to substantially influence nicotine metabolism and systemic exposure. Therefore, these results support the incorporation of CYP2A6 enzyme kinetics into the hepatic clearance model as a mechanistic strategy that could be adapted for future population or genotype‐specific simulations.

Mechanistic inhalation modeling of nicotine is a valuable approach but presents challenges since it is not feasible to directly measure regional lung deposition or absorption through in vitro or clinical sampling. Moreover, in vivo systemic pharmacokinetic data alone may not provide sufficient data to infer site‐specific deposition or absorption patterns in the lung. To address this, our model used a physiology‐informed approach to estimate regional and total nicotine absorption by leveraging published data and anatomical detail. As described earlier, we employed the PCAT model to represent the respiratory tract, dividing it into four main regions: extra‐thoracic (ET2), thoracic (BB), bronchiolar (bb), and alveolar‐interstitial (AI). These compartments correspond anatomically to the posterior nasal passage, pharynx, and mouth (ET2); the trachea and bronchi (BB); the bronchiolar tree up to the terminal bronchioles (bb); and the respiratory bronchioles up to the alveoli (AI), as described by Belete and Msganaw Shiferaw (2023). This regional framework allowed us to systematically assign deposition fractions from published studies such as Rostami et al. (2022), into our own user‐defined model, offering a more standardized approach for integrating diverse dosimetry data. Furthermore, the disposition of nicotine to settle in one compartment of the lungs versus another is dictated, in part, by swallowing from the extra‐thoracic (ET2) region. The ASF model allows us to scale the effective permeability (*P*_eff_) of different regions of the gastrointestinal tract so that we may predict how a drug was absorbed enterically. This aspect of our model also needed supplementation as the default coefficients, C1‐C4, were used as placeholders and did not reflect the specific characteristics of nicotine absorption from swallowed portions of the dose. To improve model accuracy, these coefficients were iteratively adjusted based on literature‐reported values and observed systemic pharmacokinetics from clinical studies. In doing so, we have integrated both pulmonary and enteric contributions to systemic nicotine exposure in our model.

To simulate nicotine absorption from smoking and e‐cigarette use, 100% absorption and 0% exhalation was assumed due to the short time intervals and exposure duration. For our nasal spray model, an exhalation rate of 5% was used after optimization (Supporting Information S1: Table S3). This is a rather conservative approach and may not fully reflect actual conditions, leading to an overestimation of nicotine's bioavailability. To better mimic individual conditions, future models may incorporate experimentally derived parameters that capture both partial absorption and variable exhalation rates due to differences in inhalation topography. Although the appropriate exhalation rate could theoretically be estimated considering cases where the liquid spray exits from the front of the nose, such exhalation dynamics are rarely quantified in literature, highlighting the need for further empirical investigation.

Beyond modeling absorption and exhalation, accounting for variability in nicotine metabolism amongst individuals is crucial for capturing differences in systemic exposure. E‐cigarette experienced and naive users have different rates of nicotine clearance, which is largely reliant upon the activity of the CYP2A6 enzyme (Yingst et al. 2019; Benowitz et al. 2006). Regular users of tobacco typically clear nicotine faster because regular exposure to nicotine induces the production of these enzymes. Our model used a standardized clearance across a wide variety of cohort populations and demonstrated satisfactory correlation between observed and predicted values. With minor adjustment of clearance, the model can be easily adapted to simulate the differences found in regular and naïve users of nicotine. Similarly, by changing the population profile in GastroPlus, the model can be used to simulate differences with regard to variations in age, gender, genetic polymorphism, and more. Future research should aim to integrate more personalized data to improve model accuracy. Larger and more diverse datasets in regards to demographic and disease specific physiology, as well as changes due to genotypic and phenotypic expression would serve to make the model more reliable and comprehensive.

The developed nicotine PBPK model provides a comprehensive platform for predicting systemic exposure various administration routes. By simulating concentration–time profiles under different dosing regimens, the model could contribute to dose optimization to achieve target plasma levels without excessive exposure. Although the concentrations associated with adverse effects were not available for quantitative assessment in this study, the model framework could be utilized to estimate safety margins once such data are available. This would allow evaluation of nicotine exposure under different dosing or product‐use scenarios, particularly for sensitive populations such as nonsmokers or pregnant women. Furthermore, the mechanistic framework of this model could apply to diverse exposure scenarios thereby providing a scientific basis for evaluating various nicotine formulations and informing regulatory and translational decision‐making related to nicotine exposure risks.

Nevertheless, the closely aligned plasma concentration profiles between our simulated and predicted data including that of intravenous infusion, bolus, traditional smoking, nasal spray and e‐cigarette administration support the model's suitability in predicting health‐related outcomes across various smoking scenarios. The results of our validation support the model's potential use in future studies and nicotine research and development.

## 5 Conclusion

In this study, we have developed a PBPK model that is capable of predicting the fate of nicotine following several routes of administration. Incorporation of more diverse and personalized data would serve to enhance its utility, making it a promising asset in the field of nicotine pharmacokinetic research. Application of such PBPK modeling and simulations not only enhance our understanding of nicotine pharmacokinetics but may also play an important role in public health efforts, regulatory science, and the further development of nicotine‐related therapies.

## Funding

This research was funded by the Food and Drug Administration (FDA) Center for Tobacco Products contract 75F40122C00146.

## Ethics Statement

The authors have nothing to report.

## Consent

The authors have nothing to report.

## Conflicts of Interest

The authors declare no conflicts of interest.

## Disclaimer

The findings and conclusions in this report are those of the authors and do not necessarily represent the official position of the Food and Drug Administration.

## Supporting information


Supporting Information S1

## Data Availability

The data that support the findings of this study are available from the corresponding author upon reasonable request.
