# Supplementary material for: Development of a Physiologically Based Pharmacokinetic (PBPK) Simulation Model for Nicotine
Source: Biopharm Drug Dispos. 2025 Dec 20;46(5-6):209–20. doi: 10.1002/bdd.70018 (PMC12757703; doi:10.1002/bdd.70018)
Supplement: Supplementary file 1 — Supporting Information S1 [file BDD-46-209-s001.docx]

**Supplementary material**

**
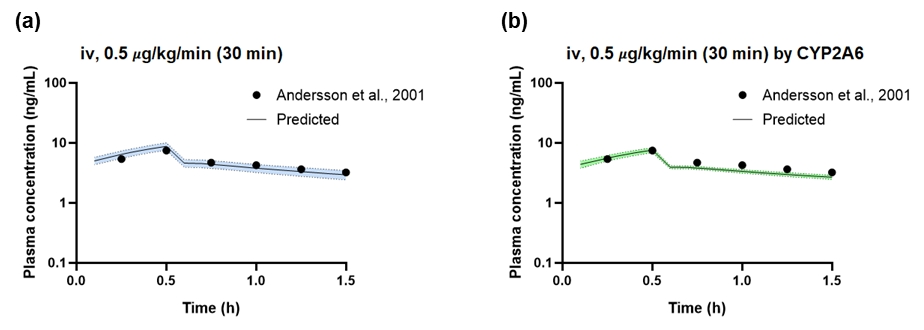
**

**Fig S1.** Plasma concentration-time profiles after iv infusion of nicotine using (a) current model (Module 1) and (b) CYP2A6 enzyme kinetic model (Module 2). iv: intravenous.

**Table S1** Clinical trials that informed the development and validation of the PBPK simulation for nicotine^*^.

| **Dose** | **Time** | **N** | **Age (year)** | **Weight (kg)** | **BMI (kg/m^2^)** | **Reference** |
| --- | --- | --- | --- | --- | --- | --- |
| ***Intravenous*** |  |  |  |  |  |  |
| 0.5 𝜇g/kg/min | 30 min | 11 | 20 – 32 | / | 20.2 – 25.3 | Andersson and Arner 2021 |
| 2 𝜇g/kg/min | 30 min | 14 | 21 – 34 | / | / | Benowitz et al. 1982 |
| 0.87 mg/h | 24 h | 14 | 27 – 64 | 56.2 - 104 | / | Benowitz et al. 1991 |
| 5.1 mg | 30 min | 12 | / | / | / | Gourlay and Benowitz 1997 |
| 2.5 𝜇g/kg multiple | 30 min | 8 | 22 – 43 | / | / | Porchet et al. 1988 |
| 0.2 𝜇g/kg/mL | bolus | 5 | 24 – 41 | / | / | Feyerabend et al. 1985 |
| ***Smoking*** |  |  |  |  |  |  |
| 2.4 mg | 10 min | 6 | / | / | / | Gourlay and Benowitz 1997 |
| ***Nasal spray*** |  |  |  |  |  |  |
| 0.8 mg | / | 6 | / | / | / | Gourlay and Benowitz 1997 |
| *E-cigarette* |  |  |  |  |  |  |
| 18 mg/mL | 10 puffs with a 30 sec interval | 16 | 18 - 55 | / | / | Rostami AA et al. 2022 |

^*^The default values in GastroPlus were used for the information not provided.

/, not available or nonapplicable

**Table S2** The optimized the tissue to plasma partition coefficients (K_p_) input values for the PBPK simulation of nicotine.

| **Organ / Tissue** | **Input value** |
| --- | --- |
| Hepatic Artery | 0.00 |
| Lung | 57.18 |
| Aterial Supply | 0.00 |
| Venous Return | 0.00 |
| Adipose | 2.56 |
| Muscle | 0.35 |
| Liver | 15.43 |
| ACAT Gut | 0.00 |
| Spleen | 5.25 |
| Heart | 8.53 |
| Brain | 5.96 |
| Kidney | 47.95 |
| Skin | 1.44 |
| ReproOrg | 2.44 |
| RedMarrow | 3.49 |
| YellowMarrow | 0.35 |
| Rest of Body | 5.00 |

**Table S3** Pulmonary absorption and setting after smoking, nasal spray and E-cigarette route for nicotine.

| **Parameter** | **Input value** | **Reference/comment** |
| --- | --- | --- |
| ***Nasal-Pulmonary Compartmental Absorption & Transit*** | |  |
| Lymph volume (mL) | 30 | Default |
| Total Lung Volume (mL) | 705.93 |  |
| Mean Inhalation Flow (mL/s) | 250 |  |
| Lymph Transit Time (h) | 0 |  |
| Pulm Solubility (mg/mL) | 93.3 |  |
| Vapor Diff Coeff (cm^2^/s) | 0.1 |  |
| Log Henry’s Law at 37°C (atm·m^3^/mol) | -5 |  |
| Henry’s Law Correction | 1 |  |
| ***Smoking / Nasal spray* / *E-cigarette*** | |  |
| Nose (%) | N.A. / 15 / N.A. | Rostami AA et al. 2022 |
| Extra-Thoracic (%) | 35 / 10 / 25 |  |
| Thoracic (%) | 10 / 10 / 10 |  |
| Bronchiolar (%) | 10 / 25 / 10 |  |
| Alveolar-Interstitial (%) | 45 / 35 / 55 |  |

N.A.: not applicable

**Table S4** The arithmetic mean (min~max) of observed and predicted pharmacokinetic parameters by the clearance-based model (Module 1) and the CYP2A6 enzyme kinetics-based model (Module 2) for nicotine after intravenous infusion administration.

| **Parameters** | **Observed** | **Module 1 Predicted** | **Module 2 Predicted** | **Reference** |
| --- | --- | --- | --- | --- |
| *C*_max_ (ng/mL) | 7.46 | 8.80 (7.59~11.0) | 7.61 (6.71~9.20) | Andersson and Arner. 2001 |
| *T*_max_ (h) | 0.500 | 0.500 | 0.500 |  |
| AUC_0-inf_ (ng·h/mL) | 13.2 | 13.7 (10.3~21.4) | 13.0 (11.6~16.7) |  |
| AUC_0-t_ (ng·h/mL) | 6.76 | 7.07 (5.91~8.49) | 6.17 (5.57~7.35) |  |

*C*_max_: the maximum plasma concentration; *T*_max_: the time to reach the maximum plasma concentration; AUC_0-inf_: the mean plasma areas under the curve extrapolated to infinity; AUC_0‐t_: the observed and predicted mean plasma areas under the curve from time zero to time t.

**Supplemental reference**

Andersson, K., and P. Arner. 2001. “Systemic Nicotine Stimulates Human Adipose Tissue Lipolysis through Local Cholinergic and Catecholaminergic Receptors.” *International Journal of Obesity and Related Metabolic Disorders* 25, no. 9: 1225–1232. https://doi.org/10.1038/sj.ijo.0801654.

Benowitz, N. L., P. Jacob III, R. T. Jones, and J. Rosenberg. 1982. “Interindividual Variability in the Metabolism and Cardiovascular Effects of Nicotine in Man.” *Journal of Pharmacology and Experimental Therapeutics* 221, no. 2: 368–372. PMID: 7077531.

Benowitz, N. L., K. Chan, C. P. Denaro, and P. Jacob III. 1991. “Stable Isotope Method for Studying Transdermal Drug Absorption: The Nicotine Patch.” *Clinical Pharmacology & Therapeutics* 50, no. 3: 286–293. https://doi.org/10.1038/clpt.1991.138.

Feyerabend, C., R. M. Ings, and M. A. Russell. 1985. “Nicotine Pharmacokinetics and Its Application to Intake from Smoking.” *British Journal of Clinical Pharmacology* 19, no. 2: 239–247. https://doi.org/10.1111/j.1365-2125.1985.tb04280.x.

Gourlay, S. G., and N. L. Benowitz. 1997. “Arteriovenous Differences in Plasma Concentration of Nicotine and Catecholamines and Related Cardiovascular Effects after Smoking, Nicotine Nasal Spray, and Intravenous Nicotine.” *Clinical Pharmacology & Therapeutics* 62, no. 5: 453–463. https://doi.org/10.1016/S0009-9236(97)90124-7.

Porchet, H. C., N. L. Benowitz, and L. B. Sheiner. 1988. “Pharmacodynamic Model of Tolerance: Application to Nicotine.” *Journal of Pharmacology and Experimental Therapeutics* 244, no. 1: 231–236. PMID: 3336000.

Rostami, A. A., J. L. Campbell, Y. B. Pithawalla, H. Pourhashem, R. S. Muhammad-Kah, M. A. Sarkar, J. Liu, W. J. McKinney, R. Gentry, and M. Gogova. 2022. “A Comprehensive Physiologically Based Pharmacokinetic (PBPK) Model for Nicotine in Humans from Using Nicotine-Containing Products with Different Routes of Exposure.” *Scientific Reports* 12: 1091. https://doi.org/10.1038/s41598-022-05108-y.
